# Effect of Polymer Additives on the Microstructure and Mechanical Properties of Self-Leveling Rubberised Concrete

**DOI:** 10.3390/ma15010249

**Published:** 2021-12-29

**Authors:** Weronika Kujawa, Iwona Tarach, Ewa Olewnik-Kruszkowska, Anna Rudawska

**Affiliations:** 1 Chair of Physical Chemistry and Physicochemistry of Polymers, Faculty of Chemistry, Nicolaus Copernicus University in Toruń, Gagarin 7 Street, 87-200 Toruń, Poland; 2 Selena Labs Sp. Z o.o., Pieszycka 1 Street, 58-200 Dzierżoniów, Poland; 3 Department of Production Engineering, Faculty of Mechanical Engineering, Lublin University of Technology, 20-618 Lublin, Poland; a.rudawska@pollub.pl

**Keywords:** polymer additives, rubber, cement-rubber composites, mechanical properties, microstructure

## Abstract

The materials based on concrete with an addition of rubber are well-known. The interaction between concrete components and rubber particles is in the majority cases insufficient. For this reason, different substances are introduced into concrete-rubber systems. The aim of this paper is to establish the influence of five different polymer additives, i.e., 1. an aqueous dispersion of a styrene-acrylic ester copolymer (silanised) (ASS), 2. water dispersion of styrene-acrylic copolymer (AS), 3. anionic copolymer of acrylic acid ester and styrene in the form of powder (AS.RDP), 4. water polymer dispersion produced from the vinyl acetate and ethylene monomers (EVA), 5. copolymer powder of vinyl acetate and ethylene (EVA.RDP)) on the properties of the self-leveling rubberised concrete. Scanning electron microscopy has allowed to establish the interaction between the cement paste and rubber aggregates. Moreover, the compressive strength and flexural strength of the studied materials were evaluated. The results indicate that the mechanical properties depend extensively on the type as well as the amount of the polymer additive introduced into the system.

## 1. Introduction

It is an enormous challenge to cause vulcanised rubber to degrade in the natural environment. For this reason, many scientists are looking for new possibilities to use that waste material. Currently, the most popular method of utilisation involves incineration or landfill disposal [1,2]. Current means of waste utilisation, however, are hazardous for the natural environment and human health [3,4]. Waste rubber produces air pollutants when incinerated while landfilling causes contamination of soil and groundwater.

One of the most efficient and environmentally friendly methods of recycling waste rubber is its utilisation by incorporation into concrete and cementitious materials. Rubberised concrete is a type of clean and eco-friendly composite, which allows utilisation of rubber waste. Natural aggregates, such as sand, which are fundamental components of concrete, can be partially replaced by rubber particles varying in size [5], amount [6], shape [7] and quality of surface [8]. The total number of waste rubber tires is estimated at more than 4 billion annually [9,10]. In contrast, the total number of produced concrete for the building industry equals 4.4 billion tons per year [11]. Combining these two materials provides an opportunity to utilise waste and obtain novel composites with unique properties.

In recent years scientists have paid attention to cement-rubber-based materials. Since 2014 the number of reported studies has been increasing every year. Most researchers focus on the mechanical properties of rubberised concrete, such as compressive and flexural strength, which belong to the fundamental properties of concrete. According to earlier reports, the incorporation of rubber particles into the cementitious matrix reduces the mechanical properties of concrete [2,12,13], especially its compressive strength [5,14]. It was established that the reduction in strength of rubber-cement composites corresponds to the increasing percentage of the weight of rubber, which replaces natural aggregates [15,16].

It should be mentioned that the deterioration of mechanical resistance is caused by the low stiffness of the rubber aggregates (RA) and the insufficient adhesion energy between hardened cement paste and rubber aggregates. The introduction of rubber into concrete results in a wide interfacial transition zone (ITZ) and, in effect, the rubber can easily withdraw from the matrix [17]. The hydrophobic nature of rubber, which tends to repel cement paste, is the reason for the formation of the above-mentioned zone [18]. The total porosity of rubberised concrete is higher in comparison with the mixture of natural aggregates and cement, where the cement paste permeates through rigid aggregates. During the mixing of water with the mixture of dry ingredients, the air bubbles are trapped on the RA surface and cause the formation of pores in the rubberised concrete matrix [19,20]. The low stiffness of rubber particles is responsible for high porosity inside the rubber-cement material as well as the reduction of mechanical characteristics of the obtained composites. Moreover, the soft rubber aggregates are easily deformed under load and contribute to the formation of microcracks in concrete. Formed cracks are developing rapidly in the ITZ, resulting in the general failure of the material [21].

In an aim to improve the mechanical properties of rubberised concrete, scientists have started researching on enhancing the interface strength between cement paste and rubber particles. According to literature, the methods which allow to improve mechanical properties of cement-rubber composites include: modifying the rubber particles surface by means of chemical treatment [22,23,24,25], coating the rubber surface [26], modifying rubber by applying plasma treatments [27] and introducing additives such as styrene-butadiene latex rubber and fumed silica [28,29]. Moreover, in the work of Lavagna et al. [30] the parameters of rubber that can influence the mechanical properties of concrete-based materials have been discussed. The main parameters include rubber content, granulometry of components, the presence of additives such as silica or pozzolana, water-to-cement ratio and functionalisation or pre-treatments by means of NaOH. Other popular chemical treatments of rubber presented in literature include silane coupling agent [31], H_2_O_2_, CaCl_2_, H_2_SO_4_ and a combination treatment of KMnO_4_ and NaHSO_4_ [32], calcium hydroxide, and acetic acid solutions [33], ethanol, methanol and acetone [34] or a chemical blend of 17.2% acrylic acid, 13.8% polyethylene glycol and 69% anhydrous ethanol by weight [35].

In the work of R. Nistico et al. [27] rubbermodified using four different plasma atmospheres (N_2_/H_2_, N_2_, N_2_/O_2_, O_2_) was introduced into two different cement matrices. The mechanical analysis of the obtained composites revealed that they are characterised by an increase in bending strength and low compressive strength in comparison to concrete contaning untreated rubber.

This work aims to analyse the structure and the mechanical properties of rubberised self-leveling concrete with polymer-based additives. The additives used in the study, i.e., poly (ethylene-co-vinyl acetate) and acrylic-styrene copolymer, belong to the group of the most popular polymer additives used in cement-based building materials. They are frequently introduced into cement in order to increase cohesion and improve mechanical parameters of the obtained materials. Their influence on the hydration process and mechanical properties of the cement matrix has been tested and described in the literature [36,37,38,39,40,41,42]. Moreover, it should be stressed that the selected polymeric additives are resistant to high pH, and for this reason, they do not coagulate during mixing with the cement paste. Therefore, the main objective of the presented work is to provide more information concering the effects of the polymer dispersions and redispersible powders on the mechanical properties of the cement–rubber composites. For this reason, the compressive strength and flexural strength of the studied materials were determined. The effect of the introduced polymer-based additives on the density and porosity of the obtained composites was evaluated. Furthermore, an adhesive joint between rubber aggregates and cement paste was observed by means of the scanning electron microscopy (SEM).

## 2. Materials and Methods

### 2.1. Materials

In an aim to form concrete mixtures, ordinary Portland cement (OPC), calcium aluminate cement (CAC), fine aggregates, ethylene propylene diene monomer rubber aggregates (EPDM), superplasticiser and polymer additives that differed in chemical structure and physical state were used. Cements as well as aggregates were obtained from local producers: OPC 52,5 R-NA from “Cemex” (Rudniki, Poland); CAC Górkal 40 from Górka (Trzebina, Poland); limestone from Czatkowice (Krzeszowice, Poland); anhydrous calcium sulfate from Nowy Ląd S.A. (Niwnice, Poland); sand from ZWP MOSTY Sp. z o.o., (Chęciny, Poland); rubber and EPDM aggregates from Unirubber Sp. z o.o. (Węgliniec, Poland). The properties of all of the aggregates used in the procedure, such as sand, limestone and rubber aggregates, have been depicted in Table 1.

The physical properties of the cements used in the procedure have been listed in Table 2.

Polymer additives used in this research included as follows: Acronal S 813 (BASF, Ludwigshafen, Germany)—water dispersion of a styrene-acrylic ester copolymer (silanised), Osakryl OSA A (Synthos, Oświęcim, Poland)—water dispersion of styrene-acrylic acid ester copolymer, Acronal P 5033 (BASF, Ludwigshafen, Germany)—anionic copolymer powder of acrylic acid ester and styrene, Vinnapas EP 17 (Wacker Chemie, Munich, Germany)-an aqueous, plasticiser-free polymer dispersion produced from the monomers vinyl acetate and ethylene, Vinnapas 5044N (Wacker Chemie, Germany)—a copolymer powder of vinyl acetate and ethylene. In the work the additives have been designated respectively: ASS, AS, AS.RDP, EVA and EVA.RDP. The details concerning polymer additives have been included in Table 3. In order to ensure self-leveling properties and to maintain the mixture’s proper homogeneity, a polycarboxylate-based superplasticiser, named Melflux 2651F, provided by BASF company (Germany) and a viscosity agent named Starvis 3070F, (BASF, Germany), which is a type of high molecular weight polymer additive were used.

### 2.2. Sample Preparation

**Figure 1:**
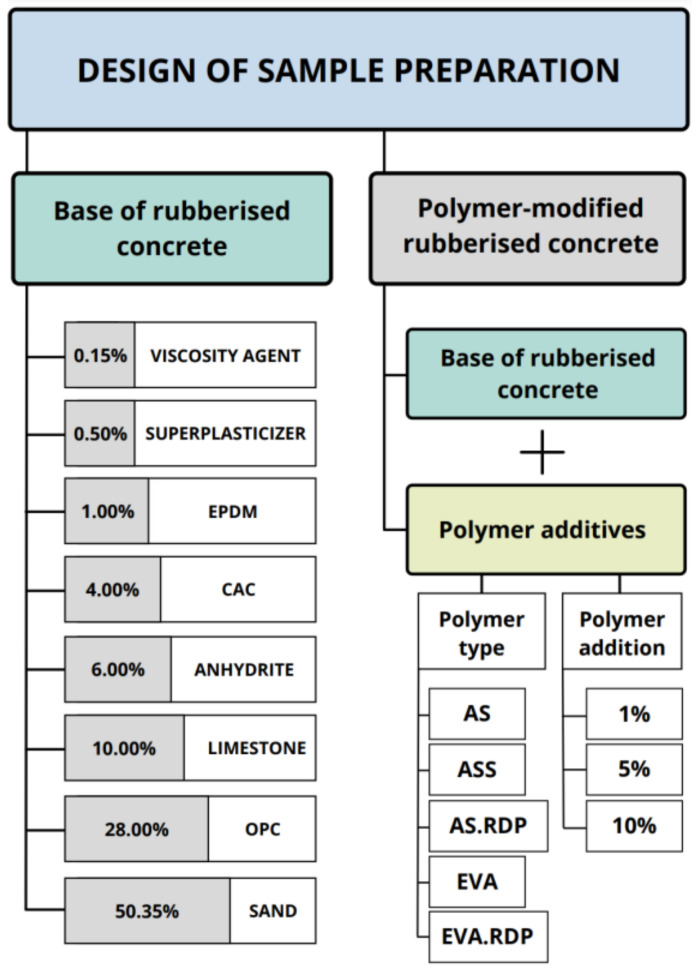
Composition of the obtained samples.

Rubberised concrete, as a reference, and 15 polymer-modified rubberised concrete types were produced at a water/cement (w/c) ratio of 0.28, with OPC content of 560 g and CAC content of 80 g. The fine sand content used in the dry cast concrete mixture amounted to 1007 g, 200 g of limestone and 10 g of superplasticiser, 3 g of viscosity agent and 120 g of anhydrite were also used. The rubber aggregates were added at a proportion of 1% to the weight of the sample. Polymer dispersions or redispersible polymer powders were incorporated into the mix with designated contents of 1%, 5% and 10%. Composition of the prepared samples is presented in Figure 1.

**Figure 2:**
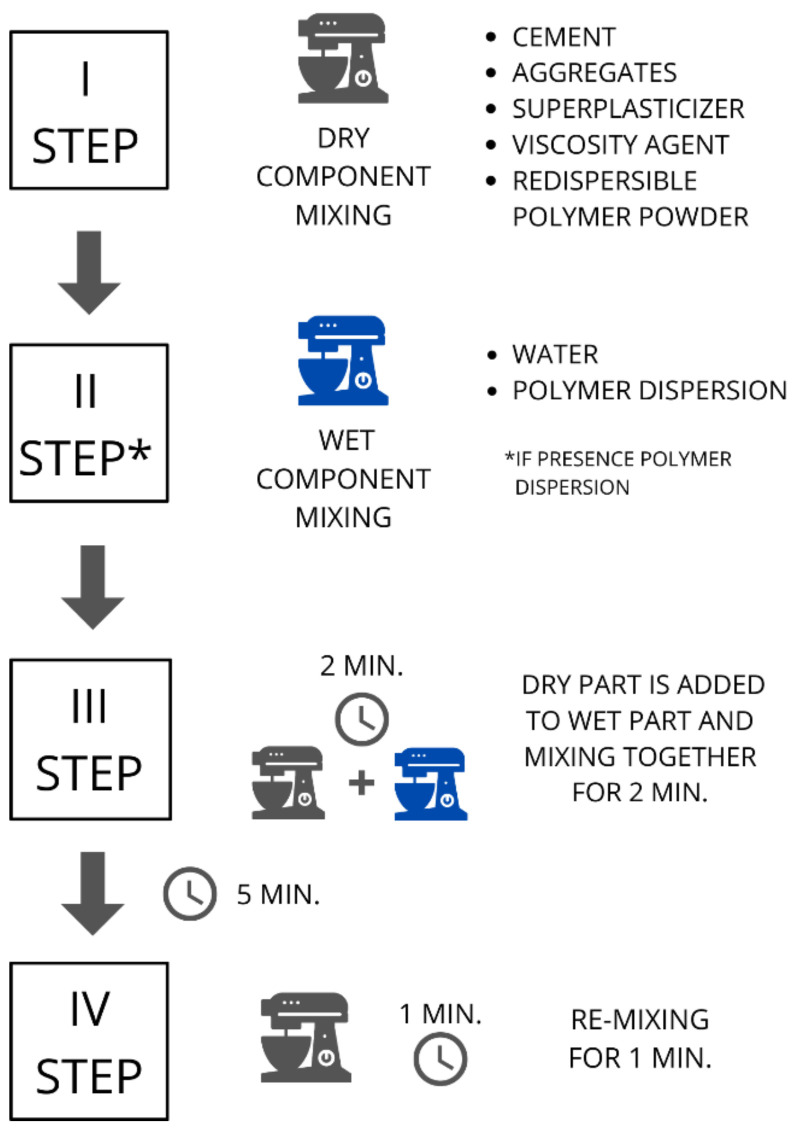
The scheme of samples formation.

The mixing process of raw materials was performed in four steps based on the standard method of mortar preparation. Initially all dry materials, including cement, aggregates, superplasticiser, viscosity agent and redispersible polymer powder (if applicable) were mixed together to prevent the agglomeration of rubber aggregates. In the second step, the water and polymer dispersion (if applicable) were mixed. Next, the dry part was added to the liquid part and mixed for 2 min. After 5 min, the mixture was re-mixed for 1 min. The concrete samples were formed in metal moulds as 160 mm × 40 mm × 40 mm cuboids. The scheme of sample formation is shown in Figure 2.

### 2.3. Workability

**Figure 3:**
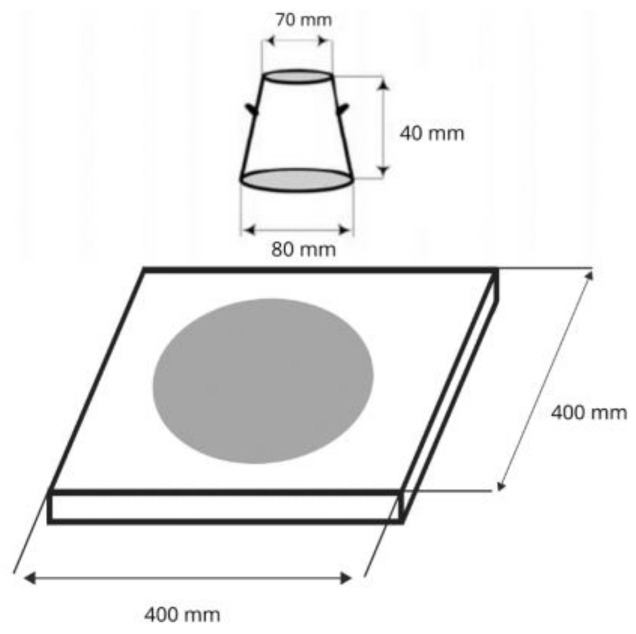
Slump flow test.

The slump test was carried out using a truncated cone (Figure 3). The slump cone was placed on a level, stable ground and filled with fresh mortar. Then the surplus fresh mortar was removed. In the next stage, the slump cone was raised vertically, allowing the concrete to flow out. On leaving the cone, the fresh material flows freely until it reaches a stable state. The self-flow properties of fresh concrete were measured in two directions after spontaneous spreading (Figure 3), and the average value of the final diameter was calculated.

### 2.4. Scanning Electron Microscopy

Microstructural studies of the rubberised self-levelling concrete with polymer additives were carried out by scanning electron microscopy (SEM). For this purpose the Quanta 3D FEG SEM/FIB (SEM, FEI Company, Hillsboro, OR, USA) scanning electron microscope with 1.2 nm resolution capability and the SE signal detection module was applied. The analysis was carried out in the variable vacuum mode. Photographs were taken at a 5000 and 25,000-fold magnification. Before the analysis, the obtained composites were sprayed with a nanometric layer of gold.

### 2.5. Density and Porosity of the Studied Materials

(a)  Particle density

The test of solid density was carried out for hardened samples of each of the tested materials at a temperature of 23 ± 2 °C using the pycnometric method in accordance with the PN-EN 1936: 2010 standard [43]. A sample of the tested material was ground and weighed (*m_s_*). The powdered sample was then placed in the pycnometer (50 mL, Isolab, Germany) and immersed in 95% ethyl alcohol solution (Avantor Performance Materials Poland S.A., Gliwice, Poland). The following measurements were taken: the mass of the pycnometer containing both the sample and alcohol (*m_p_*) and the mass of the pycnometer filled with alcohol only (*m_a_*). Mass measurements were carried out using a Radwag model WLC1/A2/C/2 balance (Radom, Poland). The measurement was performed in triplicates. The particle density (*ƍ_r_*) was calculated using the following Equation (1)

(1) $${ƍ}_{r}=\frac{m_{s}}{m_{s}+m_{a}-m_{p}}\cdot {ƍ}_{a}$$

where,

*m_s_*—the mass of the dry powdered sample, [g]
*m_a_*—the mass of the alcohol-filled pycnometer, [g]
*m_p_*—the mass of the pycnometer filled with sample and alcohol, [g]
*ƍ_a_*—alcohol density [g/cm^3^]

(b)  Bulk density

The test of solid density was carried out for hardened samples of each of the tested materials at a temperature of 23 ± 2 °C. The scales equipped with a density determination kit (Radwag, Radom, Poland) was used for the analysis. The samples were weighed in air (mass *m*_1_), in water (*m*_2_), and saturated with water in the air (*m*_3_). The measurement was performed in triplicates.

The bulk density (*ƍ_b_*) was calculated using the following Equation (2):

(2) $${ƍ}_{b}=\frac{m_{1}}{m_{3}-m_{2}}\cdot {ƍ}_{w}$$

where,

*m*_1_—the mass of dried sample in air, [g]
*m*_2_—the mass of sample in water, [g]
*m*_3_—the mass of the water-saturated sample weighed in air, [g]
*ƍ_w_*—water density at 23 °C [g/cm^3^]

(c)  Total Porosity

Total porosity *P* [%] was calculated according to the following Equation (3):

(3) $$P=\left[1-\frac{{ƍ}_{b}}{{ƍ}_{r}}\right]\cdot 100\%$$

where,

*ƍ_b_*—bulk density of the sample, [g/cm^3^]
*ƍ_r_* —particle density of sample [g/cm^3^]

(d)  Open pores volume

Open pores volume *V* [cm^3^] was calculated according to the following Equation (4)

(4) $$V=\frac{\left(m_{3}-m_{2}\right)}{{ƍ}_{w}}$$

where,

*m*_2_—the mass of sample in water, [g]
*m*_3_—the mass of the water-saturated sample weighed in air, [g]
ƍ_w_—water density at 23 °C [g/cm^3^]

(e)  Open porosity

Open porosity *P_o_* [%] was calculated according to the following Equation (5):

(5) $$P_{o}=\frac{m_{3}-m_{1}}{m_{3}-m_{2}}\cdot 100\%$$

where,

*m*_1_—the mass of dried sample in air, [g]
*m*_2_—the mass of sample in water, [g]
*m*_3_—the mass of the water-saturated sample weighed in air, [g]

### 2.6. Mechanical Properties

In an aim to perform three-point bending tests, three samples of the studied materials, measuring 40 mm × 40 mm × 160 mm, were formed. In order to determine the compressive strength, the samples were compressed after the flexural tests. Once moulded, the samples were allowed to cure in the mould for 24 h. During this period, the moulds were covered by a polyethylene film. The next step involved demoulding. The samples were kept in the laboratory at 20 ± 3 °C and 50 ± 5% relative humidity for subsequent 27 days. The specimens were cured under air conditions. The flexural and compressive strength tests were carried out according to PN EN 13892-2:2004, using testing machine 15/250 kN Multiserw Morek, Brzeźnica, Poland. About 50 N/s load was applied for the flexural test, and 2400 N/s load was applied for the compressive test.

## 3. Results and Discussion

### 3.1. Workability

**Figure 4:**
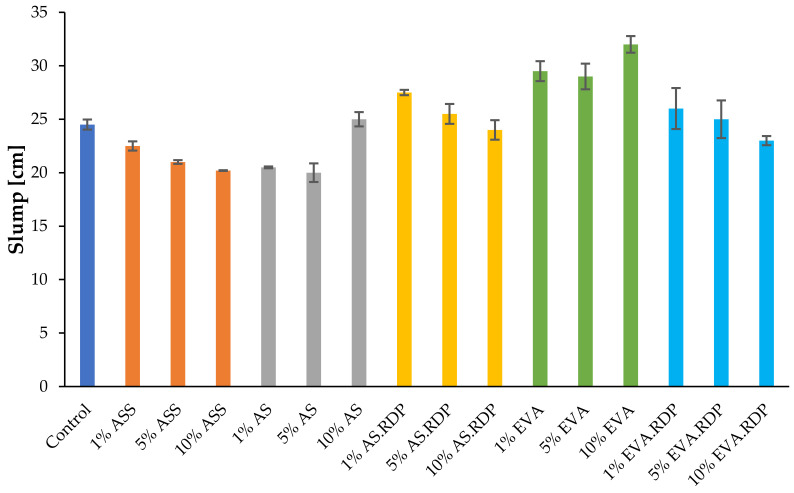
Workability of polymer-modified rubberised concrete.

Workability is the crucial factor which determines the practical application of concrete. The workability of fresh state polymer-modified rubberised concrete was determined by slump flow test, one of the primary tests for self-levelling types of concrete. All samples were analysed in order to validate the workability of the designed composites. Figure 4 shows the slump flow parameters obtained for the studied composites.

It can be clearly observed that the slump flow parameters of fresh mortar slightly increase after the addition of 1% and 5% of redispersible polymer powder additives (AS.RDP and EVA.RDP). However, an introduction of 10%wt. of redispersible polymer powders leads to the reduction in the slump parameter values compared to the control sample and the composites containing 1 and 5% wt. of AS.RDP or EVA.RDP. In literature [18] the reduction in workability is often attributed to water adsorption caused by the redispersible powder. The incorporated redispersible polymer powders form a network structure inside the cementitious matrix and need water to coalesce, which restrains the concrete slump. The slight increase in the slump parameter values can be observed at the first stage when the amount of the additive in the form of powder ranges between 1 and 5%. The same tendency was observed in the work of Schulze and Killermann [44], where composites consisting of mortar and three different redispersible powders: vinylacetate–ethylene powder, ethylene–vinylchlorid–vinyllaurate and styrene/acrylic powder were studied. The same tendency is not observed in the case of materials containing aqueous polymer dispersion (ASS, AS and EVA). In the case of composites containing EVA, an increase in polymer dispersion content caused an increase in the slump parameter values. According to literature [45] changes in the workability can be related to the increased viscosity.

The addition of ASS, however, as well as AS in most cases leads to the decrease of workability of the obtained composites in comparison to the control sample. In the work of Colak [45] it was indicated that changes in the workability can be related to the polymer structure and the interaction between the polymer additive used in the procedure and the paste. For this reason it is reasonable to assume that it is difficult to predict the slump parameter values as well as general trends.

### 3.2. Microstructure of Obtained Materials

**Figure 5:**
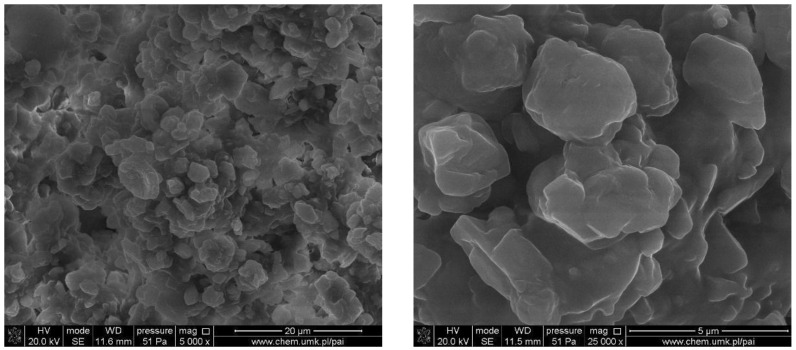
SEM microstructure of EPDM particles.

SEM was employed to observe the interface between the rubber particles, the cement matrix and polymer additives. In Figure 5 the microstructure of ethylene propylene diene monomer rubber, which is one of the components of the studied composites, is shown. It can be seen that in most cases, the particles of EPDM are characterised by an oval shape, and the size ranges from 1 to 5 µm.

**Figure 6:**
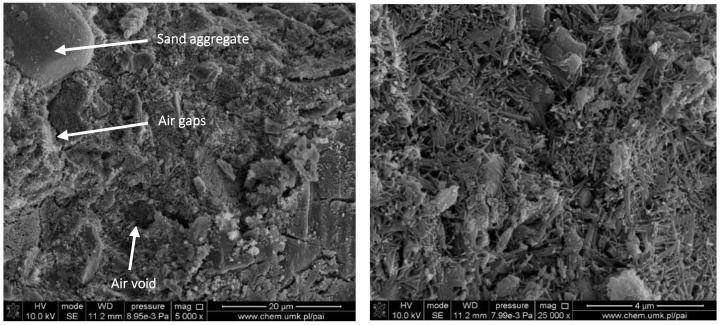
SEM microstructure of reference sample.

The SEM images of the control sample consisting of water, Portland cement, calcium aluminate cement, sand, limestone and rubber aggregates are presented in Figure 6. It is well-known that as a result of the alkali-aggregate reaction, gel shells are formed around the grain, which swell under the influence of moisture, causing stress that leads to scratches and cracks. This may be accompanied by efflorescence, stains and infiltrates.

**Figure 7:**
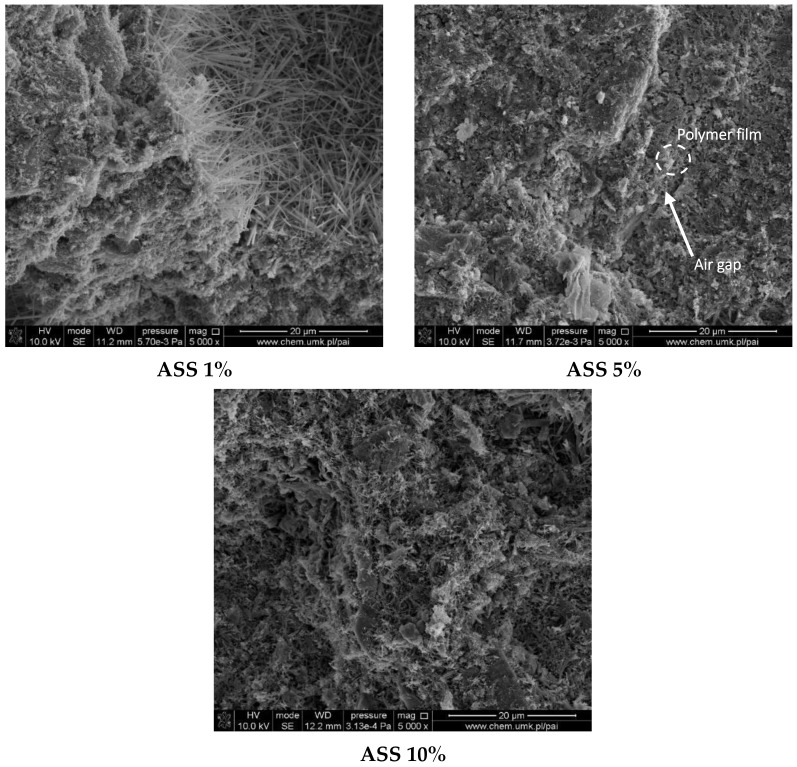
SEM microstructure of concrete with an addition of ASS (1%, 5%, 10%).

**Figure 8:**
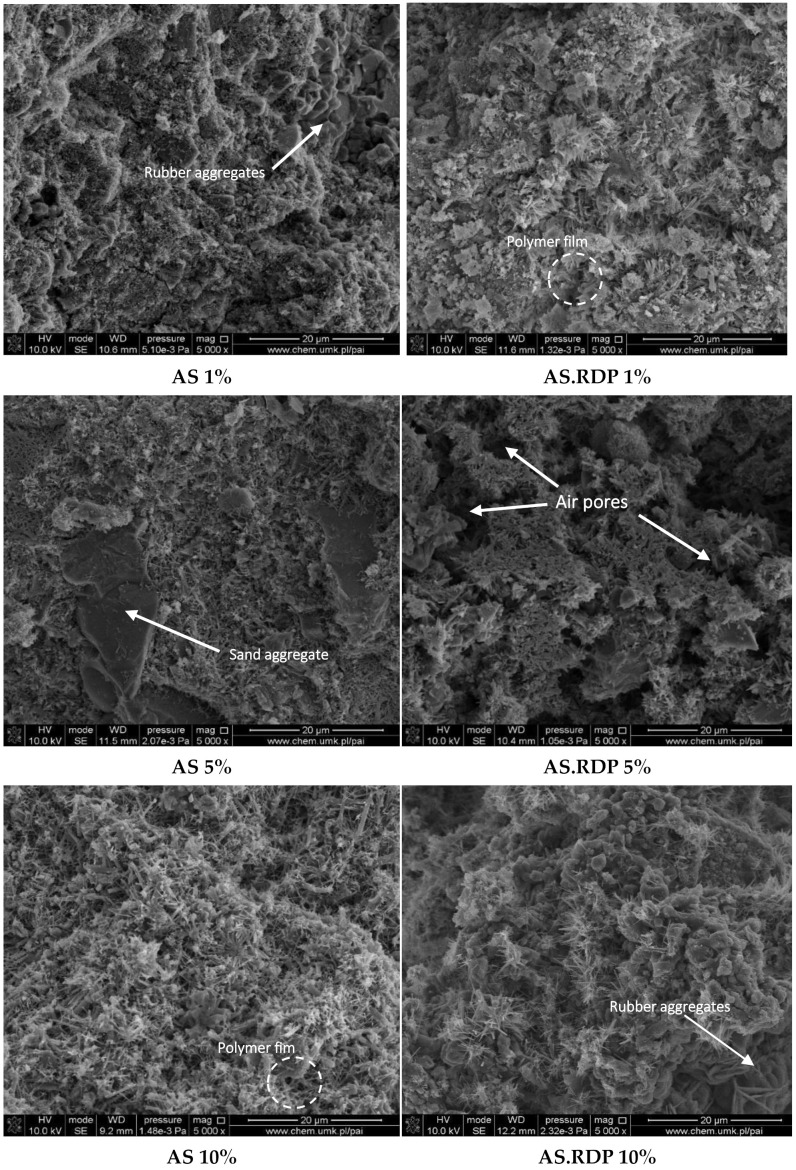
SEM microstructure of concrete with an addition of AS and AS.RDP (1%, 5%, 10%).

Subsequently, micro-cracks seen in Figure 6, chipping and detachment may cause the aggregate grains to form, reducing the durability of concrete. The symptoms of the reaction mentioned above include the formation of shells around the aggregate grains, grain breakout and a change in their volume, changes in the phase composition, the formation of micro-fissures and fissures, blooms, spots and chipping on surfaces, and consequently the destruction of concrete elements [46]. Figure 7 and Figure 8 depict the SEM images of materials with an addition of styrene-acrylic ester copolymer in the form of aqua dispersions (ASS (silanised) and AS—differ in properties) and redispersible powder (AS.RDP). In the case of samples containing silanised styrene-acrylic ester copolymer, the compatibility between the components of concrete and the introduced additive seems to be compact, especially in the case of the sample containing 1%wt of ASS. An increase in the quantity of the silanised aqua dispersions styrene-acrylic ester copolymer leads to the formation of the EPDM aggregates that may be responsible for a slight reduction in the compressive strength of concrete. In the case of materials with an addition of styrene-acrylic ester copolymer in the form of aqua dispersion (AS) or redispersible powder (AS.RDP) (Figure 8), the EPDM aggregates and air gaps in the interface of components and the cement matrix can be observed. The air gaps can result in weak bonding between cement and the introduced polymer-based additives. The same tendency was observed in the work of Gupta et al. [18]. However, it should be taken into account that the redispersible powder weakens the composite structure more significantly than the aqua dispersion.

**Figure 9:**
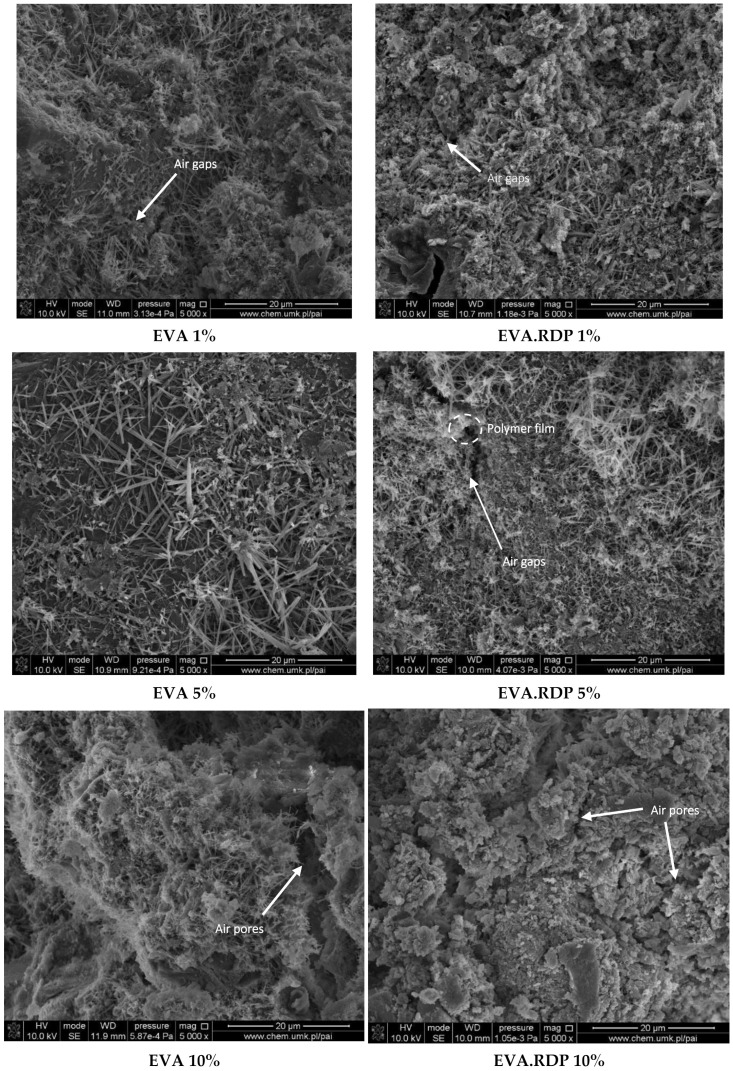
SEM microstructure of concrete with an addition of EVA and EVA.RDP (1%, 5%, 10%).

The micrographs of samples containing EVA in the form of latex and the redispersible powder are presented in Figure 9. As can be seen, the addition of EVA-based aqua dispersion allows for the formation of fibres which improves the flexural strength of the obtained materials.

The presence of the compound mentioned above leads to air retention in the internal voids that is confirmed by the increase in the porosity of the obtained composites. The same observations have been made by Silva et al. [47]. The same tendency is not observed in the case of composites filled with EVA in the form of redispersible powder. The reparation of cracks and pores of concrete-based materials in the presence of EVA redispersible powder was also observed in the literature [38,48,49,50]. Obtained results indicate that the appropriate distribution of EVA.RDP in the cement matrix allows to obtain composite with improved compressive strength compared to samples containing EVA in the form of latex.

The addition of polymer powder and polymer dispersion into concrete leads to the formation of flexible polymer films which improve adhesion and cohesion in cementitious materials. The polymer film formation is a result of the coalescence of individual latex particles after their dehydration [51]. However, it should be stressed that the interaction between cementitious and polymeric compounds is the source of considerable debate among scientists. One of the researcher groups indicates that only physical interactions take place between substrates mentioned above, while the polymeric film formed in the composite is responsible for the changes in the mechanical properties [47,52,53]. In the other works the physical and chemical interactions between polymers and cement are described and discussed [52,54,55,56]. According to the mentioned literature, introduction of polymer based-additives can lead to the formation of complex structures and cause changes in the quantity of calcium hydroxide.

Furthermore, the analysis of literature indicates that different types of polymer latexes affect the strength of the material differently [57]. Some polymer-based modifiers introduced into the concrete mix may decrease compression strength as well as flexural strength [55].

### 3.3. Density and Porosity of the Studied Materials

Density and porosity belong to the factors that influence the properties of concrete. Concrete characterised by higher density, in most cases, displays higher strength and reduced porosity [58].

**Figure 10:**
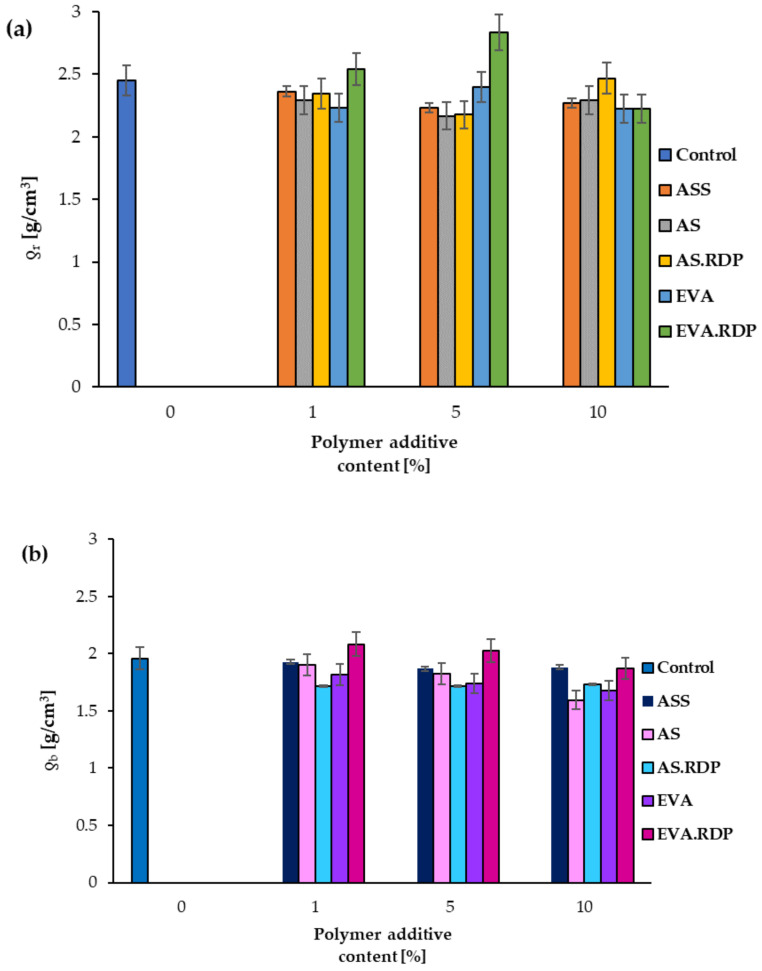
The particle (**a**) and bulk (**b**) density of the studied materials.

Reactions during concrete formation as well as inadequate compaction result cause the resulting material to consist of both solid and a pore system [18]. In an aim to establish the porosity value of concrete-based materials, different methods are applied such as mercury porosimetry [18], stereological analysis [59], nitrogen adsorption [60] or the water absorption method [61,62]. It should be stressed that some publications indicate that mercury porosimetry is an inappropriate method for the measurement of the pore size and distribution in cement-based materials [63]. In Figure 10a,b the calculated values of particle density (*ƍ_r_*) and bulk density (*ƍ_b_*) are shown respectively. The obtained results indicate that an introduction of different polymeric modifiers into concrete influences the density of the obtained composites. It should be stressed that grinding significantly affects the value of density, however, the change in the particle density values is consistent with the results obtained in the case of bulk density. Analyzing density we have to bear in mind that the change in density is influenced not only by the change in porosity but also by the properties of the polymer additive used in the process. For this reason total porosity (*P*), open pores volume (*V*) and open porosity (*Po*) were studied.

**Figure 11:**
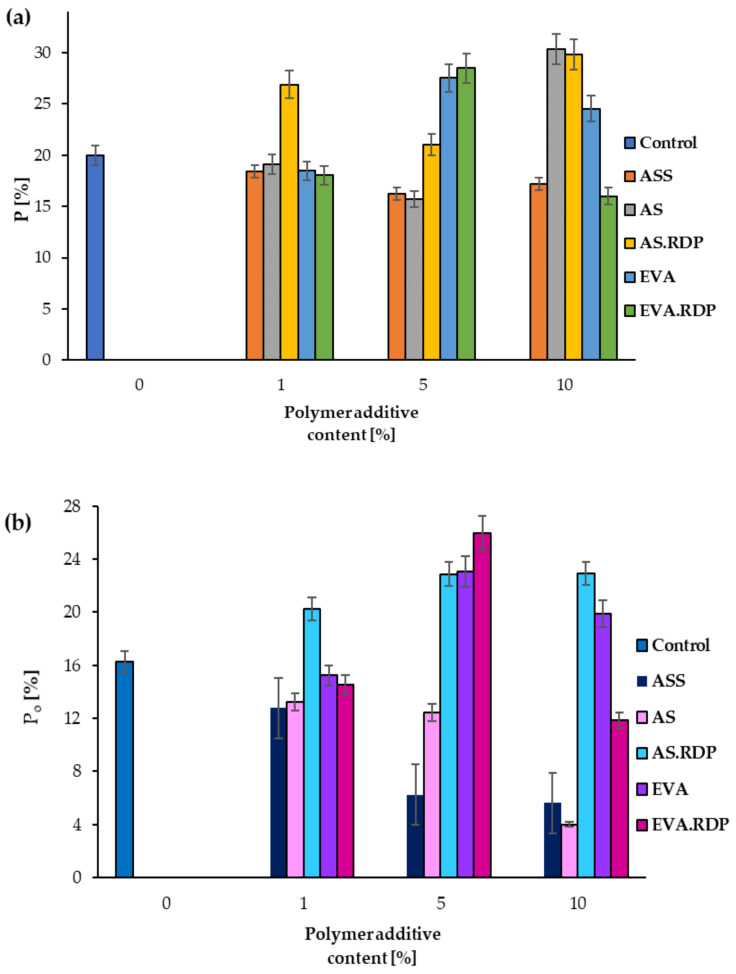
The total porosity (**a**) and open porosity (**b**) of the studied composites.

In Figure 11 the total porosity (*P*), and open porosity (*P_o_*) are shown. Based on the obtained results it has been observed that in the case samples containing anionic copolymer powder of acrylic acid ester and styrene the increase of values of total and open porosity is correlated with the reduced mechanical properties. The porosity of the ASS samples has been found to decrease with the increase in the content of the polymer additive.

**Figure 12:**
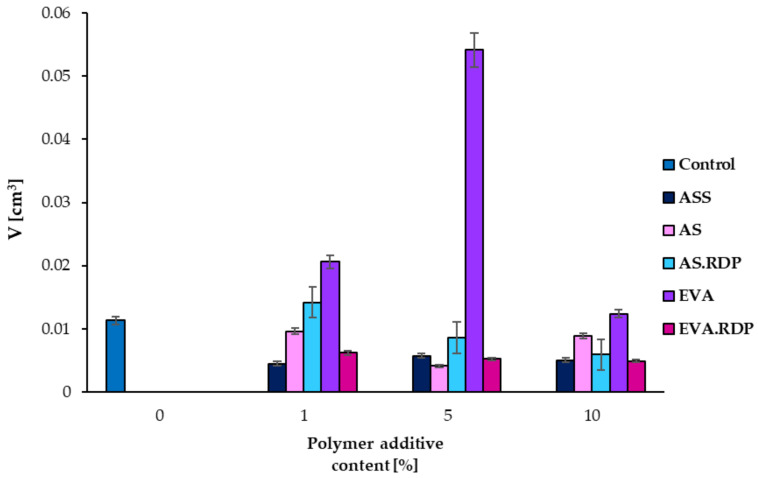
Changes in open pores volume of the studied concrete-based materials.

The data presented in Figure 12 indicate that in the studied work the introduction of polymeric additives in different forms, in most scenarios, causes a decrease of values of open pores volume. This does not, however, result in the higher values of the studied mechanical properties. Only in the case of composites containing water dispersion of a styrene-acrylic ester copolymer (silanised), the studied materials achieve higher or equal values of flexural strength and comprehension strength compared to the control sample.

### 3.4. Compressive Strength

Compressive strength is the feature of a material that allows establishing the magnitude of load that can be carried on its surface without any crack or indentation. In the case of concrete, the compressive strength depends on the quality of a particular material, quality control during the production of concrete, water–cement ratio and cement strength. In an aim to obtain desired properties, the concrete based-materials are designed to display certain mechanical properties and overall durability.

**Figure 13:**
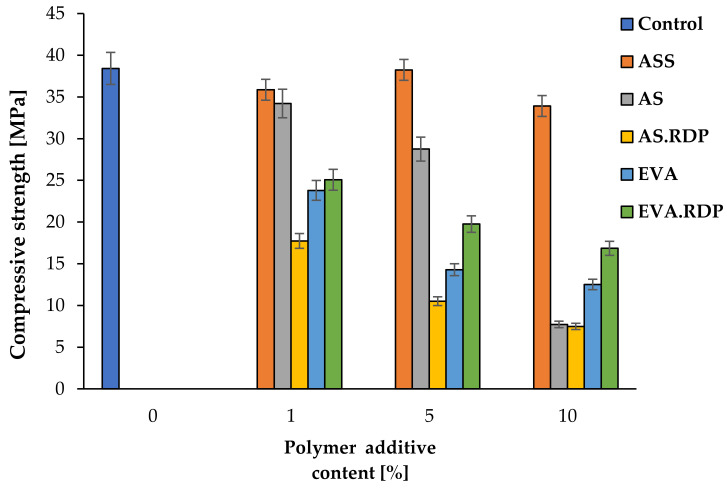
Compressive strength of the materials with polymer additives.

Figure 13 illustrates the effect of different content of the studied polymer additives on the compressive strength of the rubberised concrete-based composites. It can be clearly observed that there is a reduction in the compressive strength with an increasing additives content, except for the samples containing the ASS additive, where the highest value of compressive strength was observed in the case of the sample containing 5% of ASS. Compared to control material, the incorporation of polymer additives, both in the form of polymer dispersion and redispersible powder, into cementitious mixtures resulted in a decrease in compressive strength. Only the sample filled with 5% ASS has a similar value of compressive strength to the control sample.

The obtained results are in accordance with the literature [64,65,66], which suggests that the compressive strength decreased with the increase of polymer dispersion as well as polymer powder content in concrete. The incorporation of polymer dispersions and redispersible powders into the cementitious matrix causes a decrease in compressive strength which is caused by the lower mechanical capacity of polymers in comparison to concrete. G. Barluenga and F. Hernández-Olivares [66] have shown that the addition of polymer latexes to the concrete matrix leads to a decrease in the compressive strength (CS) of latex-modified concrete. In the work of S. Gwon et al. [65], it was established that the reduction in compressive strength is caused by the formation of a finer load-carrying pore structure with a higher polymer ratio and the formation of a monolithic structure between the polymer phases and cement hydrates. Moreover, it was indicated that CS decrease can also be the result of air entrainment by surfactants contained in the polymer powder as well as retention of water by micelles in the polymer emulsion [67].

The differences in compressive strength of polymer-modified concrete between styrene-acrylic and ethylene-vinyl acetate polymers are observed. The changes can be explained by the co-matrix formation which depends on cement hydration as well as the polymer film formation.

Many researchers have studied the interaction between polymers and cement mineral phases [54,68,69,70,71]. It is generally believed that the vinyl acetate group in the EVA polymer is hydrolyzed in an alkaline environment. The acetate anion CH_3_COO^−^ which forms during the alkaline hydrolysis of EVA in a Ca(OH)_2_ saturated cement pore solution, reacts with Ca^2+^ (originating from the dissolution of cement grains) and forms calcium acetate Ca(CH_3_COO)_2_, according to the following reaction (6) [69]. As a result of EVA-cement interaction, the Ca(OH)_2_ content decreases. Moreover, the Hadley’s grains appear inside the matrix and the ettringite crystals are well-formed [36,54].

Ca(OH)_2_ + 2CH_3_COO → Ca(CH_3_COO)_2_ + 2OH^−^ (6)

R. Wang et al. [37] have shown that the presence of styrene-acrylic copolymer in the cement paste influences cement hydration and, as a result, Ca^2+^-carboxyl complexes are formed. Su et al. [72] indicated that styrene-acrylate dispersion retards the hydration of cement. Larbi and Bijen [73] showed that Ca^2+^ ions are chelated by SAE. Subsequent studies prove that the fine-particles of latex (around 0.1 μm diameter) influence the crystallisation of calcium hydroxide. These polymer particles adhere to the surface of formed crystals [37]. All of the processes mentioned above significantly influence the observed differences in the studied compressive strength.

Moreover, it can be clearly observed, that the compressive strength values of concrete modified by means of styrene-acrylic ester polymers in dispersion form are significantly higher than in the case of composites containing redispersible powder. This is likely to result from a different structure of the polymer. The SEA powder particles are partially covered with special additives during spray drying [74]. It should also be stressed that S. Gwon et al. [65] have also observed a reduction in compressive strength after the introduction of redispersible acrylic powder into the cementitious matrix.

Comparing the values of compressive strength with the total porosity and open porosity of concrete-based materials, a correlation between these parameters can be observed. Obtained results indicate a dependency between a decrease in the compressive strength of concrete and an increase in porosity of concrete. Among the samples containing 1% of polymer additives, the lowest compressive strength has been observed in the case of 1% AS.RDP material which is characterised by the highest total porosity as well as the highest open porosity. In the case of samples containing 5% of polymer additives, a similar dependency has been observed. Moreover, it should be stressed that the sample with the lowest total porosity and open porosity had the highest compressive strength. The same tendency was observed in the case of samples with 10% of polymer additives. The composites containing 10% AS and 10% AS.RDP have achieved the same level of compressive strength. In addition, the tests revealed that these were the samples with the lowest strength.

It should be emphasised that the values of the total porosity for the 10% AS and 10% AS.RDP samples were the highest among the composites filled with 10% polymer additives and amounted to 30.34% and 29.83%, respectively. On the other hand, in the case of open porosity, the highest values of the discussed parameter were found in 10% AS.RDP (22.94%) and 10% EVA (19.89%) samples, while for the 10% AS material, the open porosity was only 4.01%. These observations suggest that the value of the compressive strength depends more significantly on the total porosity than on the open porosity.

### 3.5. Flexural Strength

**Figure 14:**
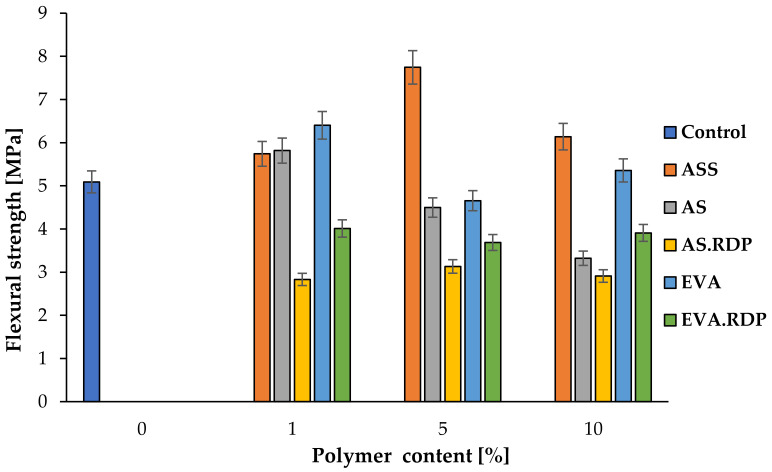
Flexural strength of the specimens with polymer addition.

Flexural strength is one of the parameters which allows to establish the tensile strength of concrete. Experimental results illustrated in Figure 10 show the effect of the polymer additives in the form of both disperse and redispersible powder on the flexural strength of rubberised concrete. As can be seen in Figure 14 in the case of six samples: 1% ASS, 1% AS, 1% EVA, 5% ASS, 10% ASS, 10% EVA the value of flexural strength is significantly higher in relation to reference material. The addition of AS to rubberised concrete caused a decrease of flexural strength with an increase in the AS content. The same tendency was observed in the work of A.F. Angelin et al. [7]. Moreover, it should be stressed that significantly lower flexural strength values were obtained in the case of samples with an addition of redispersible polymer powders, both styrene-acrylic ester, and ethylene-vinyl acetate.

Differences in flexural strength of polymer-modified rubberised concrete are caused by the changes in the concrete matrix. Enhancement samples are characterised by filled pore, especially by the ettringite which, in the case of sample ASS 1%, can be clearly seen on the SEM image (Figure 7). Moreover, it should be noted that in relation to the composite in question, the polymer matrix has been concentrated and has covered the ettringite crystals (Figure S1). Samples with a lower value of flexural strength than the reference sample are characterised by a discontinuous porous microstructure (Figure 8).

Comparison of the flexural strength with the total porosity, open porosity and pore volume of the studied concrete-based materials clearly indicates that there is no correlation between these parameters. However, it should be emphasised that Jiang and Guan [75] reported that only pores with a radius above 100 nm have a significant impact on the compressive strength. This is likely to be the reason why the obtained results are not in direct relation with mechanical properties.

## 4. Conclusions

The self-leveling rubberised concrete was modified using five different polymer additives:

- An aqueous dispersion of a styrene-acrylic ester copolymer (silanised);
- Water dispersion of styrene-acrylic copolymer;
- Anionic copolymer of acrylic acid ester and styrene in form of powder;
- Water polymer dispersion produced from the monomers vinyl acetate and ethylene;
- Copolymer powder of vinyl acetate and ethylene.

The effect of the additives on the microstructure and mechanical properties of concrete was determined. Based on the obtained results the following conclusions have been drawn:

- Introduction of polymer additives in the form of an aqueous dispersion as well as in the form of redispersive powders into the self-leveling rubberised concrete significantly influences the microstructure of the obtained materials. However, it should be stressed that silanisation of styrene-acrylic ester copolymer dispersion allows to obtain composites characterised by the lowest values of porosity and the highest values of compressive as well as flexural strength. In the case of other studied addtives there is no simple correlation between porosity and mechanical properties of the obtained concrete-based composites.
- Modification of the self-leveling rubberised concrete by means of 1% and 5% of redispersible powders increases the workability of the obtained composites, while the introduction of 10%wt. of polymeric additive leads to a reduction of studied parameter. The reduction of workability is a result of water adsorption caused by the higher amount of used redispersible powders.
- The addition of 1% aqueous polymer dispersions improved the flexural strength of the studied materials, while the admixtures in the form of powder caused the value of the studied property to decrease. The additives selected for the tests reduce the flexural strength value, with a notable exception of AS, in a larger amount (5 and 10%).

Overall, these findings suggest that the modification of the self-leveling rubberised concrete can be effective and the results of such modification significantly depend on the type and amount of the polymer additive used in the process.

## Figures and Tables

**Table 1:** Parameters of used aggregates.

Aggregate Properties	Size (mm)	Water Absorption (%)	Specific Gravity (g/dm^3^)
Sand	0.1–0.5	<0.1	1550
Limestone	<0.1	<2.0	900–1100
Rubber aggregates	<0.1	-	1.58

**Table 2:** Physical properties of cement.

Physical Properties	OPC	CAC
Initial setting (min):		
Start	181	230
End	218	270
Specific Gravity (g/cm^3^)	3.1	3.0
Blaine Fineness (cm^2^/g)	4301	3680
Compressive Strength (MPa):		
2 days	35.0	>50.0
28 days	65.3	>50.0

**Table 3:** Chemical and physical properties of polymer additives.

Properties	Acronal S 813 (ASS)	Osakryl OSA A (AS)	Acronal P 5033 (AS.RDP)	Vinnapas EP 17 (EVA)	Vinnapas 5044N (EVA.RDP)
Appearance	Aqueous dispersion	Aqueous dispersion	White powder	Aqueous dispersion	White powder
Polymer Type	styrene-acrylic ester copolymer (silanised)	styrene-acrylic acid ester copolymer	copolymer of acrylic acid ester and styrene	Ethylene-vinyl acetate	Ethylene-vinyl acetate
Viscosity (RVT 10 rpm 20 °C) [mPa·s]	100–250	1000–2000	-	2800–4800	-
Solid content [%]	49–51	48–50	>99	59–61	>98
pH	7.6–8.2	4.0–6.0	6.5–8.5	4.0–5.0	6.5–8.5
T_g_ [°C]	30.0	−2.8	−15.0	2.8	−7.1

## Data Availability

The data presented in this study are available on request from the corresponding author. The data are not publicly available due to project realisation.

## Supplementary Materials

The following supporting information can be downloaded at: https://www.mdpi.com/article/10.3390/ma15010249/s1, Figure S1. SEM images of 10% AS composite (**a**) 5000×, (**b**) 25,000×.
