# Supplementary figures and images for: Effect of Polymer Additives on the Microstructure and Mechanical Properties of Self-Leveling Rubberised Concrete
Source: Materials (Basel). 2021 Dec 29;15(1):249. doi: 10.3390/ma15010249 (PMC8746143; doi:10.3390/ma15010249)

*Supplementary materials*

**a)**

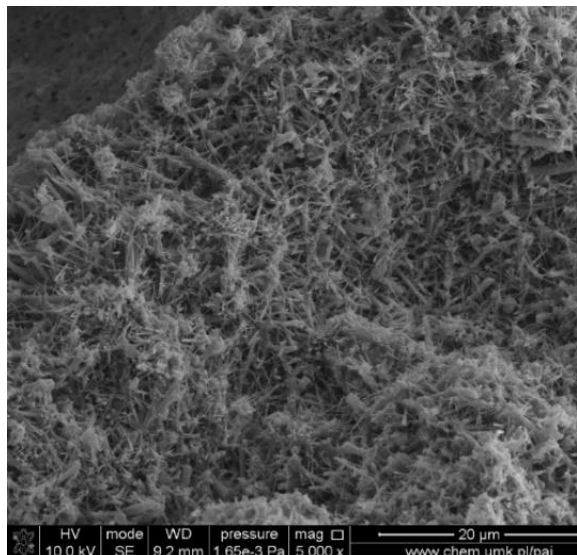

**b)**

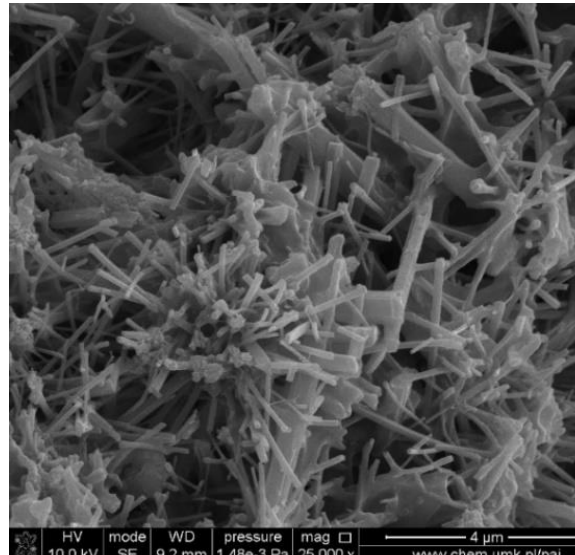

**Figure S1.** SEM images of 10% AS composite **a)** 5000x, **b)** 25000x.

Supplement: Supplementary file 1 [file materials-15-00249-s001.zip › materials-1430576-supplementary.pdf]
